# Off-Label Use of Rituximab in Patients with Different Types of Nephropathies in a Tertiary Hospital: A Retrospective Study

**DOI:** 10.3390/jcm10214941

**Published:** 2021-10-26

**Authors:** Carla Sans-Pola, Antònia Agustí, Josep Àngel Bosch, Irene Agraz, Carmen Alerany, Immaculada Danés

**Affiliations:** 1 Department of Clinical Pharmacology, Vall d’Hebron Hospital Universitari, 08035 Barcelona, Spain; csp.mir@icf.uab.cat (C.S.-P.); id@icf.uab.cat (I.D.); 2 Department of Pharmacology, Therapeutics and Toxicology, Universitat Autònoma de Barcelona, 08193 Bellaterra, Spain; 3 Immunomediated Diseases and Innovative Therapies Research Group, Vall d’Hebron Institut de Recerca (VHIR), Vall d’Hebron Hospital Universitari, 08035 Barcelona, Spain; 4 Department of Internal Medicine, Vall d’Hebron Hospital Universitari, 08035 Barcelona, Spain; jaboschg@gmail.com; 5 Department of Internal Medicine, Universitat Autònoma de Barcelona, 08193 Bellaterra, Spain; 6 Department of Nephrology, Referrer in Complex Glomerular Diseases in Adults, Vall d’Hebron Hospital Universitari, 08035 Barcelona, Spain; iagraz@vhebron.net; 7 Pharmacy Service, Vall d’Hebron Hospital Universitari, 08035 Barcelona, Spain; calerany@vhebron.net

**Keywords:** rituximab, off-label, glomerulonephritis, nephropathy, clinical pharmacology

## Abstract

Off-label use of rituximab is commonly requested for patients with resistant nephropathies. The outcomes and tolerability of rituximab in adult patients with nephropathy treated at our hospital (from 2013 to 2018) were described. Data were retrieved from electronic medical records. Response was classified as complete remission (CR), partial remission (PR), or no response (NR) according to the KDIGO criteria. A total of 89 requests were received for 61 patients. Median age was 58 years (45.9% female). Idiopathic membranous nephropathy (MN) (*n* = 30) was the most frequent indication, followed by minimal change disease (MCD) (*n* = 15) and secondary membranoproliferative glomerulonephritis (MPGN) (*n* = 12). Three patients with focal segmental glomerulosclerosis (FSGS) were included. After most treatment cycles in MN, a CR or PR was observed; median proteinuria levels significantly decreased for these patients (6000 mg/24h (IQR 3584–10,300) vs. 1468.8 (IQR 500–4604.25), *p* < 0.01). In MPGN, no response was documented after 46.7% of rituximab cycles. A CR or PR was described with the majority of rituximab cycles in MCD, with a significant decrease in proteinuria (6000 mg/24 h (IQR 4007–11,426) vs. 196.8 (IQR 100–1300), *p* = 0.013). No cycles produced a response in FSGS. Mean CD19+ B-cell decreased in all types of nephropathy (10.44% vs. 0.29%, *p* < 0.0001). Eleven patients presented infusion-related reactions, and 17 presented infectious complications. The majority of patients with MN and MCD had complete or partial responses; however, neither MPGN nor FSGS had encouraging results.

## 1. Introduction

Rituximab is a chimeric mouse/human monoclonal antibody that binds specifically to the transmembrane antigen CD20 located on B lymphocytes. It was initially approved by the European Medicines Agency in 1998 for the treatment of patients with chemoresistant stage III–IV lymphoma. Since then, its indications have broadened, and it is currently authorized for the treatment of non-Hodgkin’s lymphoma, chronic lymphocytic leukemia, rheumatoid arthritis, granulomatosis with polyangiitis and microscopic polyangiitis, and pemphigus vulgaris. However, it is also often prescribed off-label for the treatment of other indications, such as patients with resistant glomerulonephritis [1].

Off-label medicine use refers to the prescription of a drug for unapproved indications, routes of administration, or patient groups [2]. Despite it being commonly used in clinical practice, the evidence on the efficacy, safety, and tolerability of the off-label use of medicines is often scarce.

Since 2009, Spanish legislation regulates and classifies drug use in special situations, including the use of medicines in unapproved conditions, the use of unmarketed drugs, and compassionate use [3]. Taking into account that off-label use may increase the hospital spending on drugs and overall risks, the Catalan Health Service released an Instruction in 2010 to regulate its use in Catalonia [4]. A retrospective study published in 2013 described all the off-label rituximab requests received in the Vall d’Hebron University Hospital and described a high number of requests for hematologic diseases (46%), systemic connective tissue disorders (27%), and nephropathies (20%) [5]. A subsequent prospective study of patients treated with off-label drugs in five Catalan public hospitals included a total of 232 requests for 226 patients with 102 different diseases [6]. The most frequent pharmacological group was monoclonal antibodies, rituximab being the most frequent, which was used in 22 different indications in a one-year period.

So far, some case-series and small cohorts of patients with different refractory nephropathies treated with rituximab have been published [7,8,9,10,11,12].

Membranous nephropathy (MN) is the most common cause of nephrotic syndrome in non-diabetic Caucasian adults [13]. Most cases are idiopathic (primary MN), but approximately 25% of MN are secondary. Most patients with primary MN have antibodies against podocyte proteins: 70–80% have circulating anti-phospholipase A2 receptor 1 (anti-PLA2R) autoantibodies, and a small percentage of patients with secondary MN can have them too [14]. There is a tight correlation between autoantibody levels and disease activity; thus, decreasing PLA2R-antibodies titer has become an important goal [14,15,16]. The ideal treatment of patients with primary membranous nephropathy is still unknown. They have been classically treated with alkylating agents, corticosteroids, and calcineurin inhibitors; however, the treatment benefits of these medicines remain uncertain and have been associated with serious adverse events [17]. The existing evidence that B lymphocytes play a crucial role in the pathogenesis of membranous nephropathy led to the testing of rituximab as a therapeutic approach. Some observational studies have described the safety and efficacy profile of rituximab in these patients [8,9,10], and a randomized non-inferiority clinical trial concluded that rituximab was non-inferior to cyclosporine in inducing complete or partial response of proteinuria after one year [18]. Although this has since then shed some light on the use of rituximab for the treatment of MN, it is noteworthy that only 30% of patients included in the clinical trial had a history of immunosuppressive therapy use and may not have been representative of the rituximab utilization in those patients with more history of failure with other immunosuppressants. Furthermore, another clinical trial showed that treatment with corticosteroid–cyclophosphamide (Ponticelli regimen) induced remission in a significantly greater number of patients with primary MN than tacrolimus–rituximab [16].

Membranoproliferative glomerulonephritis (MPGN) is produced by deposits of antibodies that accumulate and proliferate in the basal membrane [19]. Current guidelines recommend treatment with corticosteroids and cytotoxic agents, with or without plasmapheresis, depending on the severity [17]. Rituximab has also been suggested as a treatment option for patients with primary MPGN; however, data on its use on these patients are limited to case reports and retrospective studies [11].

Minimal Change Disease (MCD) is not characterized by immune deposits and has no glomerular lesions under light microscopy [19]. Its pathogenic mechanisms primarily affect the podocyte, and, together with focal segmental glomerulosclerosis (FSGS), it is known as podocytopathy. The Kidney Disease Improving Global Outcomes (KDIGO) guidelines recommend high-dose oral corticosteroids for initial treatment of MCD and FSGS [15]. In patients with contraindications for corticosteroids, they recommend initial treatment with cyclophosphamide, calcineurin inhibitors, or mycophenolate mofetil for MCD, and calcineurin inhibitors for FSGS. MCD is thought to be T cell-mediated; thus, the role of rituximab in this disease is still unclear. Some published clinical studies have described the efficacy of rituximab in MCD in pediatric patients; however, its clinical role in adult patients remains undetermined [20,21].

Currently, predictive biomarkers for rituximab treatment efficacy are unknown. However, measurement of CD19+ B cells in blood can be used as marker of successful B cell depletion and treatment efficacy [22].

The aim of this study was to assess the rate of response and tolerability of off-label use of rituximab in patients with resistant nephropathies, as well as the clinical evolution of treated patients

## 2. Materials and Methods

A retrospective observational study of adult patients with different types of primary or secondary nephropathy treated with off-label rituximab at the Vall d’Hebron University Hospital from January 2013 to December 2017 was performed. Patients were identified from a register of the off-label rituximab requests received at the Pharmacy Service. Patients were followed-up until December 2018. The study was conducted at the Clinical Pharmacology department, in collaboration with the Nephrology and Pharmacy departments.

A review of electronic medical records was carried out to obtain demographic data, clinical data, information on the indication for rituximab use (clinical, biological, pathological, and image data according to each type of nephropathy), dosage and treatment regimen of rituximab, previous and concomitant treatments, short-term and long-term rituximab treatment outcomes, and adverse events. This information was verified by consulting the clinicians responsible for the patient’s care. Data were collected using data collection sheets and were registered into a database specifically designed for this study.

All patients treated with rituximab in the Vall d’Hebron University Hospital receive premedication intravenously before rituximab infusions, consisting of paracetamol 1 g, methylprednisolone 100 mg, and dexchlorpheniramine 5 mg, and after, they all receive prophylactic treatment with trimethoprim/sulfamethoxazole 160/800 mg to prevent infections.

Treatment response was classified as complete remission (CR), partial remission (PR), or no response (NR) according to the Kidney Disease Improving Global Outcomes (KDIGO) Guidelines for Glomerulonephritis criteria [17]. CR is defined as urinary protein excretion < 0.3 g/24 h (uPCR < 300 mg/g or < 30 mg/mmol), confirmed by two values at least 1 week apart, accompanied by a normal serum albumin concentration and a normal serum creatinine. PR refers to a urinary protein excretion < 3.5 g/24 h (uPCR < 3500 mg/g or < 350 mg/mmol) and a 50% or greater reduction from peak values, confirmed by two values at least 1 week apart, accompanied by an improvement or normalization of the serum albumin concentration and stable serum creatinine. Normal levels of protein excretion in urine are considered to be <150 mg/24 h [19]. The treatment response was assessed after each rituximab treatment cycle and for each type of nephropathy separately. Additionally, the proportion of patients who always had a CR or a PR and the proportion of those who never responded were also described. Before and after rituximab treatment CD19+ B-cell levels were only assessed for patients from 2015 to 2017 due to missing values for patients from previous years. For MN, before and after anti-PLA2R antibodies were also assessed (negative: < 14 RU/mL, borderline: 14–20 RU/mL, and positive: >20 RU/mL) [14]. Only patients from 2016 and 2017 were included in this analysis due to missing values for patients in previous years.

Adverse events were classified and assessed according to the *Medical Dictionary for Regulatory Activities* (MedDRA^®^) [23] and the algorithm of the Spanish Pharmacovigilance System [24,25]. The *International Classification of Diseases, 11th revision* (ICD-11) was used to classify medical indications for rituximab use [26].

The study was conducted according to international ethical recommendations and was approved by the local Research Ethics Committee following the national directives related to post-authorization studies.

Statistical analysis of categorical and continuous variables was performed by means of the distribution of frequencies and proportions, median, and interquartile range (IQR). Statistical differences were assessed using the Wilcoxon signed-rank test. Significance was set at a level of 0.05 and was two-tailed. The analysis was performed using RStudio 4.0.3 software (RStudio Team. RStudio: Integrated Development for R. RStudio, Inc., Boston, MA, USA).

## 3. Results

A total of 89 requests for off-label use of rituximab were received for 61 patients during the study period. All requests were approved. The median age was 58 (IQR 47.0–71.0) years, and 28 (45.9%) were female. Their baseline characteristics can be seen in Table 1. All prescribers were nephrologists. In most cases, each rituximab request or treatment cycle consisted of the administration of two doses of 1000 mg given intravenously on day 1 and day 14. Only 6.5% of cycles were adjusted to body surface (four cycles corresponding to four patients).

The most frequent indication was idiopathic membranous nephropathy (MN), with a total of 30 patients (49.2%), followed by minimal change disease (MCD) with 15 patients (24.6%) and secondary membranoproliferative glomerulonephritis (MPGN) with 12 patients (19.7%). Additionally, three patients (4.9%) with focal segmental glomerulosclerosis (FSGS) and one case of pauci-immune crescentic glomerulonephritis (PIGN), a rapidly progressive glomerulonephritis, were included. The most frequent comorbidities were hypertension and hyperlipidemia. In total, 15 patients had been previously diagnosed with a neoplasm (11 malignant; none were active at the moment of treatment with rituximab). Table 1 shows other comorbidities, previous treatments, and the median time from diagnosis to the first rituximab request for each type of nephropathy.

### 3.1. Membranous Nephropathy

Idiopathic MN was the most frequent type of nephropathy among included patients. There were two patients with secondary MN. One of them had a type 2 renal papillary carcinoma and persistent negative anti-PLA2R antibodies. The other one had a concomitant active VHC infection and positive anti-PLA2R. One of the patients with idiopathic MN was lost to follow-up after the first rituximab cycle.

There were a total of 47 rituximab requests for 30 MN patients. The median time from diagnosis to the first treatment cycle was 31.5 months (IQR 7.75–96.25), with a median time of follow-up of 33 months (IQR 19.25–51). Most patients had received some previous immunosuppressive treatment, with tacrolimus being the most frequent (19; 63.3%). Nineteen patients (63.3%) were receiving treatment with ACEIs and/or ARBs. Median baseline proteinuria levels for patients with MN were high, and their baseline renal function was slightly compromised (Table 1).

The median number of rituximab cycles was one (IQR 1–2), with a maximum of four. After rituximab treatment, the median proteinuria and serum albumin levels significantly decreased (Figure S1 and Table 2). However, for most patients this value was still over the considered normal level. Table 2 shows proteinuria, serum creatinine, and serum albumin levels before and after treatment for each type of nephropathy.

All but one patient had positive baseline levels of anti-PLA2R antibodies, with a median value of 57.65 RU/mL (IQR 14.15–105.1). After rituximab treatment, the median levels of anti-PLA2R antibodies were 2.6 RU/mL (IQR 2–5.7).

Table 3 shows the results after each rituximab treatment cycle for each type of nephropathy. After most cycles in patients with MN, a CR or PR was observed (36.2% and 40.4%, respectively). The median time from the treatment cycle to response was 2 months (IQR 1–5).

A total of 23.3% and 26.7% of patients always had a CR or PR after rituximab cycles, respectively. However, eight patients (26.7%) never responded (Table S1). Among the patients that relapsed after having responded partially or completely to rituximab, the median time from treatment to relapse was 24 months (IQR 16–31). Additional information can be found in Table S1.

A total of 22 patients (73.3%) received concomitant treatment with one or more immunosuppressive medication during the treatment with rituximab, and the same number of patients needed further therapy after rituximab, either to maintain disease remission or to treat new flares. Table 4 shows concomitant and post-rituximab treatments.

### 3.2. Membranoproliferative Glomerulonephritis

Most of the included MPGN cases were related to HCV infection and type II cryoglobulinemia. There was only one patient with idiopathic MPGN. There was a total of 15 off-label rituximab requests for 12 patients. Median time from diagnosis to the first cycle was 4 months (IQR 1–10), and median follow-up time was 26 months (IQR 9.25–63). See Table 1 for baseline characteristics.

For these patients, the median number of previous immunosuppressants was 1.5 (IQR 1–3), and the most frequently used were corticosteroids (7; 58.3%), followed by cyclophosphamide (4; 33.3%), in accordance with current clinical practice guidelines. In addition, to treat HCV infection prior to the rituximab treatment, four patients with HCV-related secondary MPGN had already received antiviral therapy, such as entecavir, ritonavir, lamivudine, ribavirin, and sofosbuvir. Median baseline proteinuria levels for patients with MPGN were high, although they appeared to be milder than for patients with MN; however, their baseline renal function appeared to be highly compromised.

The median number of rituximab cycles was one (IQR 1–2), and the patient who received the higher number of treatment cycles received a total of two. A total of 46.7% of cycles did not induce disease remission (Table 3), and there was no significant difference between before and after proteinuria levels (Table 2). Among those cycles that induced remission, the median time from treatment to response was 0.5 months (IQR 0–1).

Half of the MPGN patients never responded to rituximab (Table S1). A total of 10 patients (83.3%) received concomitant therapy, with a median number of concomitant immunosuppressants of 1.5 (IQR 1–3) (Table 4). Other therapies were also used concomitantly in some patients, such as plasmapheresis (1; 8.3%), hemodialysis (1; 8.3%), and antiviral medicines to treat the HCV infection (3; 25%). Most patients required other therapies after rituximab treatment to achieve disease remission (Table 4).

### 3.3. Minimal Change Disease

All cases of MCD were idiopathic. There was a total of 23 requests for 15 patients. One patient who received only one cycle was lost to follow-up. The median time from diagnosis to the first treatment cycle was 15.5 months (IQR 9–38.75). Median follow-up time was 24 months (IQR 17–30.5). Most patients had received prior immunosuppressant treatments. The median baseline proteinuria was high, and serum albumin levels were accordingly low. Their renal function (glomerular filtration rate and serum creatinine) was not as compromised. Other baseline characteristics can be seen in Table 1. A total of 86.7% of patients had a steroid-dependent nephrotic syndrome.

The median number of rituximab cycles was one (IQR 1–2), with a maximum of three. There was a significant decrease in before and after rituximab proteinuria and a significant increase in serum albumin levels (Table 2). As seen in Table 3 after most cycles, some response was observed (CR in 56.5% and PR in 26.1% PR). The median time from treatment cycle to response was 1 month (IQR 1–1).

A total of 66.7% of patients (*n* = 10) always had a CR after rituximab cycles, and only 13.3% of them (*n* = 2) never responded (Table S1). Among the patients who relapsed after having a PR or CR, the median time from treatment to relapse was 15 months (IQR 12.75–27).

A total of 12 patients (80%) received one or more concomitant immunosuppressants during their treatment with rituximab, with a median number of two different agents (IQR 1.5–2) (Table 4). The need for immunosuppressive treatment seemed lower after receiving rituximab (median 1; IQR 0–1.5), mainly due to a reduction in the use of corticosteroids. Treatments after rituximab therapy can be seen in Table 4.

### 3.4. Focal Segmental Glomerulosclerosis

Two patients with idiopathic FSGS and one patient with genetic FSGS, related to LMX1B gene mutation, were included. There were three requests for three patients. The median time from diagnosis to the first request was 9.5 months (IQR 7.25–11.75). Baseline proteinuria levels were very high, with accordingly low serum albumin levels, and their baseline renal function was compromised. Other baseline characteristics can be seen in Table 1.

All patients had received two or more previous immunosuppressive drugs, with a median of four (IQR 3–4), and two of them had received previous treatment with ACEIs and/or ARBs (Table 1). None of the cycles produced a treatment response (Table 3), and there were no significant differences in before and after proteinuria levels (Table 2). All patients required concomitant and post-rituximab medication (Table 4). Two of them required hemodialysis.

### 3.5. Pauci-Immune Crescentic Glomerulonephritis

The single case of rapidly progressive glomerulonephritis was a 72-year-old patient who did not respond to previous treatment with corticosteroids (Table 1). Anti-glomerular basement membrane antibodies (Anti-GBM) and antineutrophil cytoplasmic antibodies (ANCA) were both negative, and the patient was diagnosed with pauci-immune crescentic glomerulonephritis (PICGN). The patient received one rituximab cycle, but no response was achieved (Table 2, Table 3 and Table S1) and eventually required treatment with plasmapheresis and cyclophosphamide with bad results (Table 4).

### 3.6. CD19+ B-Cell Levels

From the year 2015 to 2017, 31 patients received one or more rituximab treatment cycles: 13 MN patients (41.9%), 12 MCD patients (38.7%), 4 MPGN patients (12.9%), and 2 other patients with a FSGS and a RPGN. Considering all the patients included in this period (*n* = 31), the CD19+ B cell values (in percentage) were higher before rituximab treatment than after, with mean values of 10.44% (95% confidence interval (95% CI) 7.6–14.2) and 0.29% (95% CI 0.008–1), respectively. This difference was statistically significant (*p* < 0.0001). The median time from treatment to CD19+ B cell assessment was of 1 month (IQR 1–2).

**Figure 1:**
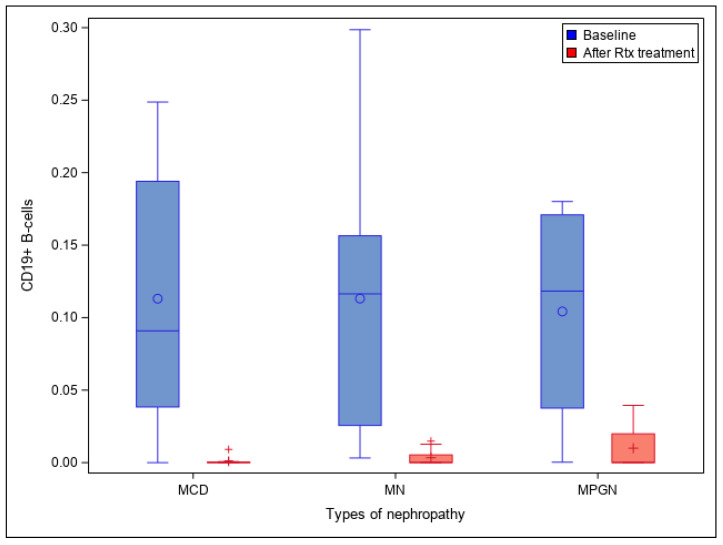
CD19+ B cell values before and after rituximab (RTX) for each of the three main types of nephropathy (MN, MCD, and MPGN). MN: membranous nephropathy. MPGN: membranoproliferative glomerulonephritis. MCD: minimal change disease. Rtx: rituximab.

All patients had a depletion of CD19+ B cells after rituximab treatment, and most (80.6%) responded to rituximab; however, six patients (19.4%) did not have a significant decrease in proteinuria levels and did not respond to the treatment. It is worth mentioning that four patients had no detectable CD19+ B cells at baseline but were treated with rituximab regardless because of the severity of their disease. They all had a complete or partial response. Figure 1 shows mean CD19+ B cell values before and after rituximab for each of the three main types of nephropathies (MN, MCD, and MPGN) in this period.

### 3.7. Adverse Events

Twenty-eight patients (45.9%) presented adverse events during the study period (Table 5). Eleven patients (18.0%) presented infusion-related adverse reactions, such as skin rash, uvular edema, rhinorrhea, sneezing, and dysphonia during the infusion. One of these patients presented a similar reaction after the first administration of ofatumumab. One patient had a serum sickness-like reaction after the rituximab administration. Other adverse events included a non-ST elevation myocardial infarction during the infusion that required coronary catheterization and stent implantation. Seventeen patients (27.9%) presented one or more infectious complications during the study period. Five patients (8.2%) did not receive any further rituximab cycles due to adverse events (three infusion reactions, one serum sickness-like, and one myocardial infarction).

## 4. Discussion

The results of this study show that the off-label use of rituximab among our patient cohort was mainly for the treatment of idiopathic membranous nephropathy (MN), minimal change disease (MCD), and membranoproliferative glomerulonephritis (MPGN). Some complete and partial responses after rituximab treatment were achieved in patients with MN and MCD; however, treatment response in patients with MPGN or focal segmental glomerulosclerosis (FSGS) was scarce. Most patients had received immunosuppressive treatments before the first rituximab request.

Available evidence for using rituximab to treat these diseases is variable and still scarce, mainly based on observational studies and case-series. In patients with MN, some observational studies have described the safety and effectiveness profile of rituximab [8,9,10,27]. A randomized non-inferiority clinical trial (MENTOR) published in 2018, which included 130 patients with membranous nephropathy and compared rituximab and cyclosporine for the treatment of these patients, concluded that rituximab was non-inferior to cyclosporine in inducing complete or partial remission of proteinuria at 12 months and was superior in maintaining proteinuria remission up to 24 months [18]. Sixty percent of patients of the rituximab group had a complete or partial remission at 24 months, after two cycles of rituximab administered at the beginning of the study and after 6 months in the case of partial response. Patients included in our study had a greater previous exposure to other immunosuppressants before rituximab use than those included in the MENTOR study, suggesting that could probably have a more severe or prolonged disease. Another randomized open-label controlled clinical trial (STARMEN) concluded that treatment with a traditional Ponticelli regimen induced remission in a significantly greater number of patients than a sequential treatment of tacrolimus and rituximab [16]. It is worth noting that patients who had received previous treatment with corticosteroids, other immunosuppressive agents, or any other biologic agent some time before screening, and those who were nonresponsive to previous immunosuppressants, were excluded from this trial. This is far from the reality of patients with MN treated with rituximab in clinical practice. Thus, once again, patients included in our study had a greater previous exposure to other immunosuppressants before rituximab use. This suggests that the results from currently available clinical trials may not be applicable in patients with a more severe disease. Additionally, while it is true that a cyclophosphamide-based regime mostly results in rapid control of the disease, it is worth noting that they are sometimes avoided or discontinued in clinical practice due to its long-term toxicity. It is speculated by some authors that this toxicity could be decreased by reducing glucocorticoid administration or by using less cyclophosphamide but in combination with rituximab [28].

Despite this, our results show that most of the patients with MN had either a complete or a partial response after rituximab treatment, and in half of them, some response was always maintained during follow-up. Among those patients who relapsed after a response, the median time from treatment to relapse was 24 months. There were significant differences in before and after proteinuria levels and a decrease in anti-PLA2R antibody levels. In the STARMEN trial, anti-PLA2R levels showed a significant decrease in both groups, and early immunologic response was followed by clinical remission in most patients, which confirms the usefulness of anti-PLA2R monitoring in this disease [16]. Another trial shows that anti-PLA2R levels are early markers of rituximab efficacy, whereas the effect on proteinuria remission appears after 6 months, suggesting that they should be included in criteria for remission [15]. There are currently other ongoing clinical trials evaluating the use of rituximab in MN (clinicaltrials.gov, NCT04154787, NCT03949855, NCT04743739, NCT03018535, NCT03880643, NCT03804359, and NCT00977977).

Some studies showed partial and complete responses in idiopathic MPGN, but rituximab was not effective in patients with complement-mediated C3 glomerulonephritis and dense deposit disease [11]. The results of these studies have been variable, and it is still difficult to draw any conclusions. There are no results from randomized clinical trials evaluating rituximab in MPGN. Our results show that half of the patients with MPGN never responded to rituximab, and there were no differences between the before and after proteinuria levels. However, more than half of patients with some response after rituximab treatment did not relapse during the follow-up period. Most of these patients had MPGN related to HCV infection and received specific antiviral treatment after rituximab, which could have contributed to the maintenance of the therapeutic response during the follow-up period. Due to the limited number of patients in our study, these results should be confirmed with larger studies.

As for MCD and FSGS, Hansrivijit et al. conducted a systematic review and meta-analysis that included a total of 221 adult patients with MCD or FSGS, and the results indicated that rituximab may be considered as an additional treatment to the standard therapy for these patients [29]. In this study, 53.6% of patients with FSGS and 80.3% with MCD achieved remission, with disease recurring in 47.3% and 35.9%, respectively. These results must be interpreted taking into account a possible publication bias. Recently, a retrospective study assessing the use of rituximab for refractory or relapsed FSGS or MCD in 25 patients has been completed, but no results have been published yet (clinicaltrials.gov, NCT04369183), and there is currently an ongoing phase 3 clinical trial that aims to enroll 40 patients (clinicaltrials.gov, NCT03298698). In our study, most patients with MCD always had a complete or partial response after rituximab treatment, and most patients achieved a complete or partial response after rituximab cycles. More than half of them maintained the good response during study follow-up period. The fact that the number of immunosuppressive agents seemed to decrease after having received treatment with rituximab suggests a better outcome in these patients during the study period. In a similar way to MN, MCD patients had a significant decrease in proteinuria levels after treatment compared with baseline. The limited available evidence in patients with FSGS suggests that rituximab does not achieve great results, and similarly, the results in our patients with FSGS were not good. However, a recently published retrospective study that included 21 patients with FSGS suggests that rituximab significantly reduces the number of relapses [30]. The small number of patients treated in our center does not allow us to draw any conclusions.

The interpretation of these results should be carried out bearing in mind that the patients were refractory to or dependent on other treatments. Additionally, the fact that some patients were receiving concomitant immunosuppressant treatments and that the majority needed further treatment after rituximab treatment to maintain disease remission or to treat new flares, including plasmapheresis and hemodialysis, needs to be considered.

Our CD19+ B cell count analysis showed that there were statistically significant differences between the values before and after rituximab treatment and that most of the patients included in this analysis had a decrease in CD19 levels and a decreased proteinuria. However, we do not have enough evidence to conclude that those patients with no changes in CD19 levels did not respond to treatment with rituximab. Some of our patients with no CD19+ B cells at baseline responded to rituximab. Similar cases of treatment success despite the absence of circulating B cells have been described, and some authors suggest a possible role of CD20+ T cells in the pathogenesis of MCD [29]. The study by Fervenza et al. shows that, after rituximab, the CD19+ counts remained low at 12 months. Thus, a residual therapeutic effect of rituximab beyond this time period cannot be ruled out [18]. Nonetheless, in three previous studies, CD19+ counts at 12 months showed no relation to proteinuria response [15,31,32], and there is a lack of information on CD19 counts from other clinical trials [16,18].

The existence of anti-rituximab antibodies that could neutralize rituximab B cell cytotoxicity and consequently impact the clinical outcome of patients has been suggested as a possible explanation for the course of those patients that respond to the first cycles of rituximab but stop achieving responses for subsequent cycles. Some authors suggest that fully human anti-CD20, such as ofatumumab, could be a therapeutic alternative for these patients [33].

Adverse events identified in our study were similar to previously published observational case series [8,10,11,12,34]. Infections were the most frequently described adverse events in our study, with a higher rate than in clinical trials and meta-analysis [18,31,33,34,35]. However, it is worth noting that most of our patients received other immunosuppressant drugs concomitantly or after treatment with rituximab, which can have an impact on the incidence of infections. A total of 71% of patients in the rituximab group included in the MENTOR trial presented an adverse event during the trial study period, and 25% presented an infusion-related reaction (18% in our study) [18].

This study has some limitations. Firstly, it is an observational study with a retrospective design and without a control group; thus, results might be subject to bias. For the same reason, some results were missing in clinical records, and missing values had to be handled in some analyses. In addition, the group of diseases included, although they are all nephropathies, had different pathogenetic mechanisms involved, and the characteristics of the patients were also different. Consequently, this implies a smaller number of patients in each group. In addition, only one center was included in our study; therefore, our results can be affected by a selection bias and cannot be extrapolated to other hospitals in other geographical areas. Treatment strategies may have changed during the study period, and rituximab is probably used more frequently in some patients with MN in the later years. The main strengths of our study are, on the one hand, that the participating center is a third-level hospital with all medical and surgical specialties and a high level of complexity; and, on the other hand, all patients were followed by the same two nephrologists, both of whom are experts in glomerular disease and assessed patients following the same criteria. Additionally, each patient was normally assessed by the same nephrology specialist in each visit. This reduces the variability in the assessment of the treatment response. Furthermore, all rituximab requests were assessed and approved by the drug and therapeutics committee, which guarantees an additional thorough evaluation of each case.

## 5. Conclusions

Among our patients, membranous nephropathy, minimal change disease, and membranoproliferative glomerulonephritis were the main indications for off-label use of rituximab in nephropathies. Some short-term partial and complete response was achieved for patients with membranous nephropathy and minimal change disease, but neither membranoproliferative glomerulonephritis nor focal segmental glomerulosclerosis had encouraging results in our study. Data from future prospective studies and clinical trials may provide useful information on the results of rituximab treatment in patients with these diseases to further improve clinical practice and off-label prescribing decisions.

## Figures and Tables

**Table 1:** Baseline clinical characteristics of patients.

Type of GN *n* (%)	MN 30 (49.2)	MPGN 12 (19.7)	MCD 15 (24.6)	FSGS 3 (4.9)	PICGN 1 (1.6)
Age (median (IQR)) years	61 (45–71)	58 (53–66)	53 (43–7)	52 (48–60)	72
Sex Women, *n* (%) Men, *n* (%)	12 (40) 18 (60)	7 (58.3) 5 (41.7)	7 (46.6) 8 (53.3)	2 (66.7) 1 (33.3)	0 1 (100)
Tobacco smoking, *n* (%)	11 (36.7)	8 (66.6)	6 (40.0)	1 (33.3)	1 (100)
Alcohol consumption, *n* (%)	4 (13.3)	3 (25.0)	0	0	1 (100)
Comorbidities, *n* (%) Hypertension Hyperlipidemia Obesity Type 2 diabetes Hyperuricemia Neoplasms Autoimmune diseases Hepatitis C infection Hepatitis B infection Type II cryoglobulinemia	16 (53.3) 17 (56.7) 4 (13.3) 5 (16.7) 9 (30.0) 6 (20.0) 2 (6.7) 1 (3.3) 1 (3.3) 0	8 (66.7) 4 (33.3) 1 (8.3) 3 (25.0) 0 6 (50.0) 5 (41.7) 9 (75.0) 3 (25.0) 9 (75.0)	6 (40.0) 3 (20.0) 4 (26.7) 1 (6.7) 1 (6.7) 2 (13.3) 4 (26.7) 1 (6.7) 0 0	1 (33.3) 2 (66.7) 1 (33.3) 1 (33.3) 0 0 2 (66.7) 0 0 0	1 (100) 0 0 0 0 1 (100) 1 (100) 0 0 0
Proteinuria (median (IQR)) mg/24 h	6700 (3584–12,800)	1856 (771.5–3423.8)	6000 (4300–11,800)	8078 (4418.8–10,313.2)	N/D
Serum creatinine (median (IQR)) mg/dL	1.11 (0.89–1.83)	2.06 (1.21–2.93)	0.98 (0.78–1.52)	1.9 (1.37–2.03)	7.26
Serum albumin (median (IQR)) g/dL	3.08 (2.4–3.6)	3.13 (3.03–3.56)	2.9 (2.26–3.1)	2.97 (2.89–3.39)	N/D
Glomerular filtration rate (median (IQR)) mL/min/1.73 m^2^	75 (59–89)	29 (20.5–48.5)	77.5 (52–90)	40 (33–63.9)	7
Months from diagnosis to first RTX cycle (median (IQR))	31.5 (7.75–96.25)	4 (1–10)	15.5 (9–38.75)	9.50 (7.25–11.75)	0
No. of previous immunosuppressive treatments (median (IQR))	1 (1–2)	1.5 (1–3)	2 (1.5–2)	4 (3–4)	1
Previous immunosuppressants *n* (%) Corticosteroids Tacrolimus Mycophenolate mofetil Other	10 (33.3) 19 (63.3) 4 (13.3) 5 (16.7)	7 (58.3) 3 (25.0) 1 (8.3) 5 (41.7)	13 (86.7) 10 (66.7) 2 (13.3) 3 (20.0)	3 (100) 2 (66.7) 2 (66.7) 2 (66.7)	1 (100) 0 0 0
Other					
ACEIs or ARBs *n* (%)	19 (63.3)	3 (2.5)	10 (66.7)	2 (66.7)	0

MN: membranous nephropathy. MPGN: membranoproliferative glomerulonephritis. MCD: minimal change disease. FSGS: focal segmental glomerulosclerosis. RTX: rituximab. N/D: No data available. IQR: interquartile range.

**Table 2:** Proteinuria, serum creatinine, and serum albumin values before and after rituximab treatment for the three main types of nephropathies.

	MN			MPGN			MCD		
	Before	After	*p*	Before	After	*p*	Before	After	*p*
Proteinuria (median (IQR)) mg/24 h	6000 (3584–10,300)	1468.8 (500–4604.25)	<0.01	1856 (771.5–3423.8)	1000 (846–6036)	0.59	6000 (4007–11,426)	196.8 (100–1300)	0.013
Serum creatinine (median (IQR)) mg/dL	1.01 (0.89–1.26)	1.05 (0.91–1.33)	0.58	1.7 (1.21–2.78)	1.55 (1.18–3.08)	0.64	1.05 (0.79–1.53)	0.88 (0.74–1.16)	0.56
Serum albumin (median (IQR)) g/dL	3.34 (2.528–3.653)	3.85 (3–4.095)	0.002	3.13 (3.1–3.56)	3.305 (3.028–4.033)	0.33	2.9 (2.26–3.25)	4 (3.6–4.22)	0.003

N: membranous nephropathy. MPGN: membranoproliferative glomerulonephritis. MCD: minimal change disease. IQR: interquartile range.

**Table 3:** Observed outcome after each rituximab treatment cycle.

Type of GN	MN	MPGN	MCD	FSGS	PICGN
No. of RTX treatment cycles, *n*	47	15	23	3	1
Outcome after RTX treatment cycles: CR, *n* (%) PR, *n* (%) NR, *n* (%) Unknown, *n* (%)	17 (36.2) 19 (40.4) 10 (21.3) 1 (2.1) ^a^	4 (26.7) 4 (26.7) 7 (46.7) 0	13 (56.5) 6 (26.1) 3 (13.0) 1 (4.3) ^a^	0 0 3 (100) 0	0 0 1 (100) 0

MN: membranous nephropathy. MPGN: membranoproliferative glomerulonephritis. MCD: minimal change disease. FSGS: focal segmental glomerulosclerosis. CR: complete response. PR: partial response. NR: no response. RTX: rituximab. ^a^ Patients lost to follow-up.

**Table 4:** Concomitant and post-RTX treatment.

Type of GN *n* (%)	MN 30 (49.2)	MPGN 12 (19.7)	MCD 15 (24.6)	FSGS 3 (4.9)	PICGN 1 (1.6)
No. of concomitant immunosuppressive treatments (median (IQR))	1 (1–2)	1.5 (1–3)	2 (1.5–2)	4 (3–4)	1
Concomitant immunosuppressants, *n* (%) Corticosteroids Tacrolimus Mycophenolate mofetil Cyclosporine A	4 (13.3) 16 (53.3) 2 (6.7) 2 (6.7)	3 (25.0) 2 (16.7) 3 (25.0) 1 (8.3)	7 (46.7) 4 (26.7) 3 (20.0) 1 (6.7)	2 (66.7%) 1 (33.3) 0 1 (33.3)	1 (100) 0 0 0
No. of post-RTX immunosuppressive treatments (median (IQR))	1 (0.25–2)	2.5 (2–3)	1 (0–1.5)	1 (1–2.5)	2
Post-RTX immunosuppressants, *n* (%) Corticosteroids Tacrolimus Mycophenolate mofetil Other	5 (16.7) 18 (60.0) 3 (10.0) 5 (16.7)	2 (16.7) 5 (41.7) 2 (16.7) 1 (8.3)	4 (26.7) 4 (26.7) 4 (26.7) 0	1 (33.3) 1 (33.3) 0 1 (33.3)	0 0 0 1 (100)
Post-RTX plasmapheresis, *n* (%)	0	4 (33.3)	0	0	1 (100)
Post-RTX hemodialysis, *n* (%)	2 (6.7)	2 (16.7)	2 (13.3)	2 (66.7)	0

MN: membranous nephropathy. MPGN: membranoproliferative glomerulonephritis. MCD: minimal change disease. FSGS: focal segmental glomerulosclerosis. IQR: interquartile range.

**Table 5:** Summary of adverse events.

Adverse Event	*n* (%)
*Any adverse event*	28 (45.9)
*Adverse events*^a^:	
Infections	24 (39.3)
Sepsis without septic shock	5 (8.2)
Urinary tract infection	4 (6.6)
Sepsis with septic shock	3 (4.9)
Lower respiratory infection	3 (4.9)
Bacterial cellulitis	2 (3.3)
Cytomegaloviral disease	2 (3.3)
Dental abscess	1 (1.6)
Postoperative wound infection	1 (1.6)
Pyothorax	1 (1.6)
Gastroenteritis due to *Campylobacter*	1 (1.6)
Intestinal infections due to *Clostridioides difficile*	1 (1.6)
Infusion-related reaction	11 (18.0)
Serum sickness-like reaction	1 (1.6)
ST-elevation myocardial infarction	1 (1.6)

^a^ 28 patients presented 37 adverse events during the study period.

## Data Availability

The data presented in this study are available on request from the corresponding author.

## Supplementary Materials

The following are available online at https://www.mdpi.com/article/10.3390/jcm10214941/s1, Table S1: Observed patient outcomes for each type of nephropathy, Figure S1: Proteinuria levels before and after rituximab (RTX) treatment for each type of nephropathy.
