# Supplementary material for: Off-Label Use of Rituximab in Patients with Different Types of Nephropathies in a Tertiary Hospital: A Retrospective Study
Source: J Clin Med. 2021 Oct 26;10(21):4941. doi: 10.3390/jcm10214941 (PMC8584949; doi:10.3390/jcm10214941)
Supplement: Supplementary file 1 [file jcm-10-04941-s001.zip › jcm-1405795-supplementary.pdf]

Table S1. Observed patient outcomes for each type of nephropathy

**Type of GN**	**MN** (*n*=30)	**MPGN** (*n*=12)	**MCD** (*n*=15)	**FSGS** (*n*=3)	**Other** (*n*=1)
Always CR, *n* (%)	7 (23.3%)	2 (16.7%)	10 (66.7%)	0	0
Always PR, *n* (%)	8 (26.7%)	4 (33.3%)	1 (6.7%)	0	0
Always NR, *n* (%)	8 (26.7%)	6 (50.0%)	2 (13.3%)	3 (100%)	1 (100%)
Other, *n* (%)	7 (23.3%) [a]	0	2 (13.3%) [b]	0	0

MN: membranous nephropathy. MPGN: membranoproliferative glomerulonephritis. MCD: minimal change disease. FSGS: focal segmental glomerulosclerosis. CR: complete response. PR: partial response. NR: no response.

[a] 6 patients presented a CR after the first cycle, but PR after subsequent cycles. 1 patient presented a PR after the first cycle but did not respond to the second cycle.
[b] 1 patient presented a PR after the first cycle, but a CR after the second, and 1 patient did not respond to the first cycle but presented a PR to the second.

Figure S1. Proteinuria levels before and after rituximab (RTX) treatment for each type of nephropathy

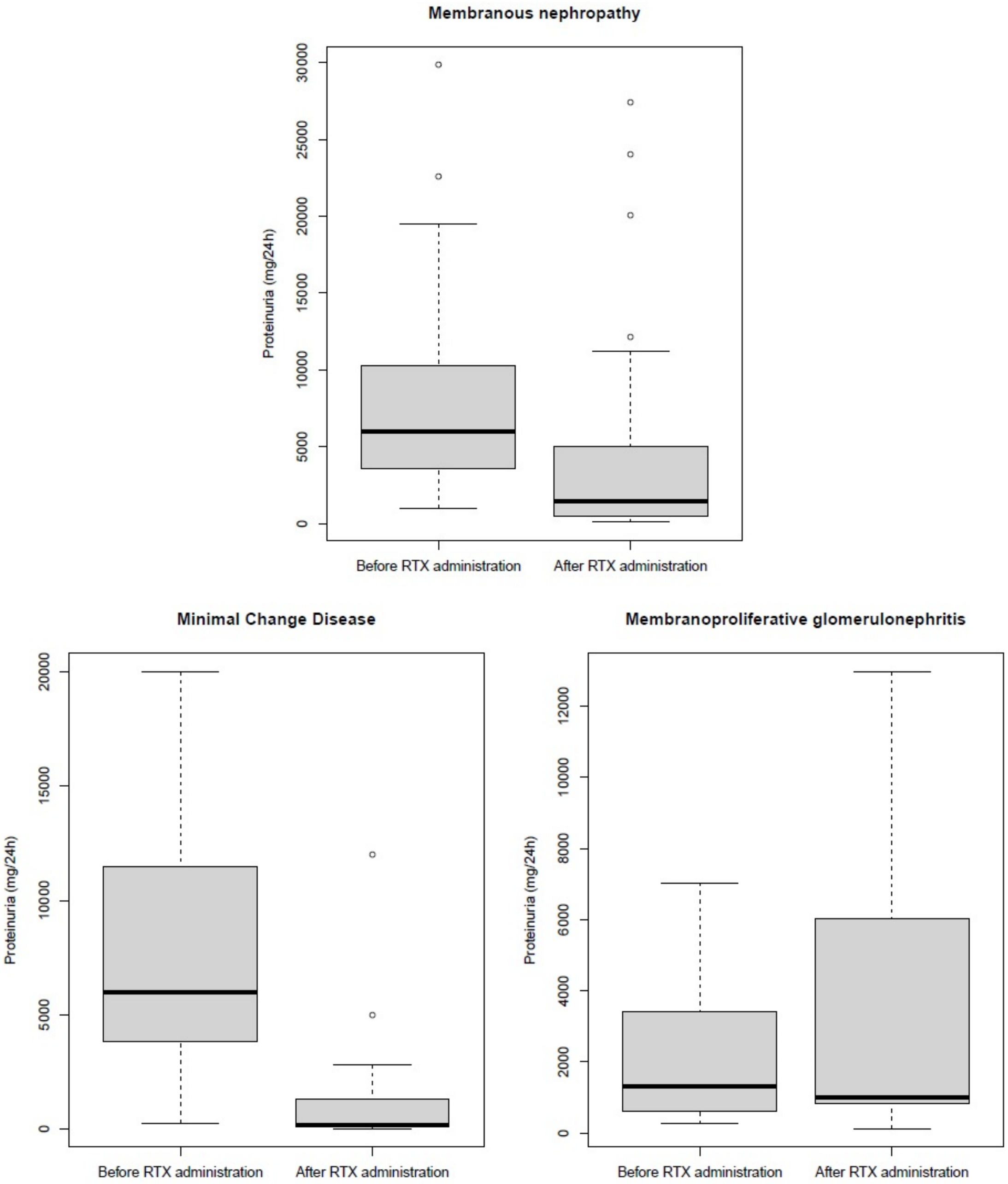

RTX: rituximab
